# A Real Time Method Based on Deep Learning for Reconstructing Holographic Acoustic Fields from Phased Transducer Arrays

**DOI:** 10.3390/mi14061108

**Published:** 2023-05-24

**Authors:** Shuai Wang, Xuewei Wang, Fucheng You, Yang Li, Han Xiao

**Affiliations:** College of Information Engineering, Beijing Institute of Graphic Communication, Beijing 102627, China; 15102552156@163.com (S.W.);

**Keywords:** holographic acoustic field, machine learning, phased transducer array, attentional mechanisms, ultrasound

## Abstract

Phased transducer arrays (PTA) can control ultrasonic waves to produce a holographic acoustic field. However, obtaining the phase of the corresponding PTA from a given holographic acoustic field is an inverse propagation problem, which is a mathematically unsolvable nonlinear system. Most of the existing methods use iterative methods, which are complex and time-consuming. To better solve this problem, this paper proposed a novel method based on deep learning to reconstruct the holographic sound field from PTA. For the imbalance and randomness of the focal point distribution in the holographic acoustic field, we constructed a novel neural network structure incorporating attention mechanisms to focus on useful focal point information in the holographic sound field. The results showed that the transducer phase distribution obtained from the neural network fully supports the PTA to generate the corresponding holographic sound field, and the simulated holographic sound field can be reconstructed with high efficiency and quality. The method proposed in this paper has the advantage of real-time performance that is difficult to achieve by traditional iterative methods and has the advantage of higher accuracy compared with the novel AcousNet methods.

## 1. Introduction

PTA is a device that uses acoustic transducers as ultrasonic sound-generating units. The form of PTA is generally several transducers arranged into a two-dimensional rectangular array. During operation, each transducer emits acoustic waves with the same amplitude [1], and the acoustic phase of the signal emitted by each transducer is controlled independently using electrical signals so that multiple groups of acoustic waves are dynamically superimposed in the acoustic field to produce a specific shape of acoustic radiation potential field. The holographic acoustic field is an acoustic field in which both sound pressure intensity and phase information are recorded. PTA generates holographic acoustic fields by using the principle of superimposed interference of acoustic waves. During the generation of the holographic acoustic field, each transducer that emits an acoustic wave frequency is kept constant, and a signal delay is used to change the phase difference between the individual transducers [2]. The ultrasonic waves emitted by the PTA superimpose interference in the target space to achieve the focusing, deflection, and deflective focusing of the acoustic waves [3].

PTA generates a holographic acoustic field that can be used for specific operations, such as traveling wave tweezers based on acoustic radiation forces that can drive particles toward acoustic pressure nodes or anti-node positions [4]. This allows us to create one or more focal points to non-contact rotate or move the particles [5,6]. In contrast to other non-contact energy drives, such as magnetic drives [7] and optical drives [8], acoustic waves have the ability to penetrate through thick and opaque media relative to light waves and are highly applicable relative to magnetic field manipulation as it does not require the material to be magnetic. In addition, acoustic waves have the advantages of good biocompatibility [9], low power consumption, and high output force, so acoustic drives show great potential in various applications [10].

The process of generating the holographic acoustic field from the PTA is straightforward, but solving the phase distribution of each transducer in the PTA backwards from the holographic acoustic field is difficult, and it is a mathematically unsolvable nonlinear problem. Low-quality holographic acoustic fields may lead to inaccurate focal points and affect the accuracy. Accurately calculating the phase distribution of each transducer in the PTA with a known target holographic acoustic field is still a challenge. The traditional methods of solving this problem are generally through iterative methods such as iterative angular spectrum methods [11] and projection iterative methods. The iterative angular spectrum algorithm (IASA) [12] is suitable for solving phase calculations in two-dimensional acoustic field images, i.e., acoustic holograms [13]. However, IASA does not normalize the transducer-emitted acoustic wave amplitude, so it cannot be directly used for PTA. In 2018, Marzo and Drinkwater et al. [14] proposed the iterative back propagation (IBP) method, which is applicable to solve the phase problem in 3D holographic acoustic field generation. However, the IBP algorithm cannot be mathematically proven to be convergent, and its iterative process is too time-consuming to be applied in real time, so it is not applicable to the reconstruction of complex holographic acoustic field generation. An emerging approach to solve this problem is based on machine learning, such as the AcousNet [15] method proposed by Chengxi Zhong et al. at the University of Science and Technology of Shanghai in 2021. The phase distribution of the PTA is directly predicted by training the VGG-based network model to learn a large number of inverse mapping relations. The unique advantage of this method is that it has a real-time performance unmatched by traditional methods and achieves a high degree of reconstruction of the holographic acoustic field. However, the AcousNet method does not take into account the randomness of the focal information in the acoustic field and the strength of the sound pressure distribution and therefore suffers from low accuracy.

To further promote a wider practical application of non-contact operations based on holographic acoustic fields and to solve the problems of the above reconstruction methods, in this paper, we propose a deep learning (DL) network framework with higher prediction accuracy to solve this nonlinear inverse mapping problem quickly and efficiently, which can achieve a real-time reconstruction of the holographic acoustic field. The remaining parts of this paper are organized as follows. In Section 2, we build a physical model of the holographic acoustic field generated by the PTA. In Section 3, we propose a DL framework for generating holographic acoustic fields from PTA and experimentally validate the high-accuracy prediction of the phase distribution of transducers in PTA by this neural network. In Section 4, we describe the dataset preparation and preprocessing. In Section 5, we experimentally validate the good reconstruction performance of the proposed neural network for the target holographic acoustic field.

## 2. Physical Model

In this section, the physical model of the holographic acoustic field generated by the PTA is elaborated. First, two coordinate systems are established, the coordinates (*m*, *n*) are used to represent the location of the transducer in the PTA and a Cartesian coordinate (*x*, *y*, *z*) with the origin **O** located at the center of the PTA is used to represent the sampling points. The positions of the transducers are arranged in the form of a two-dimensional array located in the **XOY** plane. The initial phase of the transducer-emitted acoustic wave can be indexed as $\varphi_{mn}$ and lies in the range of [0, 2π]. The parameters of the PTA are operating frequency, number of array elements, spacing, and area, respectively.

**Figure 1:**
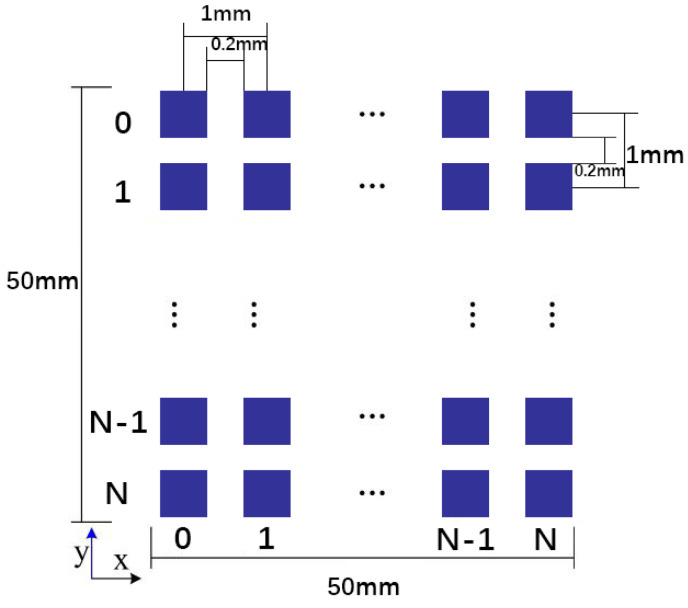
Dimensional geometry diagram of PTA.

In this study, we use an IBP algorithm by Asier Marzo et al. [14] to generate the required dataset for manipulating particles in an air medium using PTA systems. The algorithm sets the frequency of the acoustic waves to 40 kHz, which we also use as the voltage excitation signal of the PTA. To design our array, we consider that Seki Inoue et al. have used single-sided arrays of up to 996 transducers and double-sided arrays of 1992 transducers to levitate large particles [16]. Therefore, we use an array consisting of 50 × 50 transducers with a center-to-center distance of 1 mm and a side length of 0.8 mm for each square transducer. The total area of the array is 50 × 50 ${\mathrm{mm}}^{2}$. The dimensional geometry of the PTA is illustrated in Figure 1.

**Figure 2:**
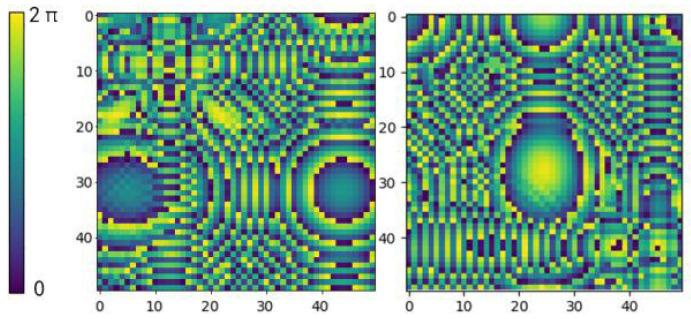
Phase distribution of acoustic waves emitted by the transducer on the PTA.

The phase of the acoustic wave emitted by each transducer in the PTA is controlled independently, and its phase distribution can be regarded as an image with a pixel value of $R_{n\times n}$. The two images in Figure 2 visualize the distribution of transducer phase values in the PTA. The image uses different colors to represent different phase values, where each pixel value represents the phase value of one transducer. The same color is used for points with the same phase value, and the acoustic wave is periodic so that 0 and 2π represent the same phase value. The holographic acoustic field region is above the **XOY** plane, and the compound pressure at a location point (*x*, *y*, *z*) in the acoustic field region is labeled as *p*(*x*, *y*, *z*), as shown in Equation (1), where $A_{x,y,z}$ represents the sound pressure amplitude at the point (*x*, *y*, *z*).

(1) $$p\left(x,y,z\right)=A_{x,y,z}e^{j\left(x,y,z\right)}$$

**Figure 3:**
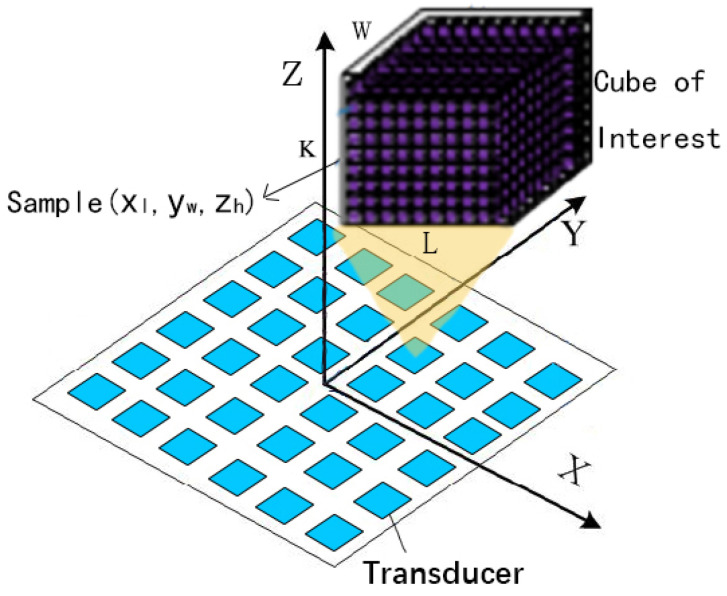
Schematic diagram of the holographic acoustic field generated by the PTA composed of rectangular transducer elements.

To accurately simulate the holographic acoustic field by the model, the holographic acoustic field needs to be effectively quantified and reorganized. Discrete sample points are first collected using a cube of interest (COI) to encapsulate the target acoustic field region. Then, the COI is discretized into smaller sub-cubes with the number of samples, and then sample points ($x_{l},y_{w},z_{h}$) are randomly selected from each sub-cube. The shape of the neural network input tensor depends on how the COI is discretized. To ensure that the input structure of the neural network remains constant, the target region needs to always yield a determined number of samples *L* × *W* × *K* for holographic acoustic fields of different workspace sizes and locations. To accurately describe the acoustic field to achieve finer control, we followed a study by Zhong Chengxi et al. on holographic acoustic field quantification [15], where the number of sample points is three or four times the number of transducers. In this paper, we set *L* = *W* = *K* = 20 and obtained 8000 samples from a given COI so that they can be processed as input to the neural network for training. These discrete samples can build 8000 equations based on the forward propagation formula, which is basically sufficient to calculate the phase of the transducer in the PTA in the actual sound field reconstruction. A schematic diagram of the holographic acoustic field generated by the PTA is shown in Figure 3.

In this paper, **H**∈$R_{n\times n\times n}$ is used to describe the spatially complex holographic acoustic field distribution and **I∈**$R_{n\times n}$ to describe the distribution of transducers on the PTA. The amount and location of the focal information in the holographic acoustic field depends on the phase of the acoustic waves emitted by the different transducers. In the PTA, the excitation signal of each transducer is controlled independently so that the acoustic waves interfere and superimpose in the spatial region above the PTA to produce a certain acoustic field [17]. The forward mapping **F** of ultrasound waves propagating forward from the PTA to produce a holographic acoustic field is described as:

**F**:  **I** (*m*, *n*) -> **H** (*x*, *y*, *z*) (2)

where *m*, *n* ≤ *N*, $\left|x\right|$, $\left|y\right|$ ≤ *S*1, 0 < *z ≤ S*2

In order to produce the highly controllable expected holographic acoustic field in practical applications, it is necessary to obtain the transducer phase distribution required to reconstruct the holographic acoustic field. That is, the phase of the acoustic waves emitted by the transducer needs to be solved. This inverse mapping **F’** to obtain the phase distribution of the PTA from the holographic acoustic field is described as:

**F**:  **H** (*x*, *y*, *z*) -> **I** (*m*, *n*) (3)

where *m*, *n* ≤ *N*, $\left|x\right|$, $\left|y\right|$ ≤ *S*1, 0 < *z ≤ S*2, where (*m*, *n*) is the coordinate position of the transducer, (*x*, *y*, *z*) is the coordinate position of the sampling point in the acoustic field, *N* is the custom PTA size, *S*1 and *S*2 are the custom acoustic field sizes. Equation (2) above is the forward propagation modeling, which can be solved directly with the acoustic theory model to obtain the sound pressure information in the holographic acoustic field. Equation (3) is the inverse propagation modeling, which is difficult to solve mathematically for the phase information in the PTA due to the high nonlinearity. Therefore, we propose a deep learning-based approach to solve the back-propagation problem from the holographic acoustic field to the PTA. Detailed information about the dataset preparation and the neural network architecture is given below.

## 3. Methodology

We propose a residual-based convolutional neural network to learn the inverse mapping **F’** defined by Equation (3). The implementation process is as follows: the information of the sampled points in the holographic acoustic field is input to the network to predict the phase of the transducer in the PTA, the oss function is calculated by comparing the difference between the true phase mean and the predicted phase mean, and the gradient descent algorithm is used for optimization to finally obtain the transducer phase that satisfies the error requirement. The proposed neural network architecture and the design of the loss function are described in detail in this section.

### 3.1. Framework of the Proposed Model

In the process of generating the holographic acoustic field, different weak acoustic pressure signals are randomly distributed into the acoustic field region due to the non-uniform distribution of acoustic pressure and the background noise caused by external interference sound waves. When such acoustic field signals are superimposed, the difference between the weaker part of the sound pressure signal and the background will be reduced, making the information of the weak focus difficult to be detected accurately, thus affecting the accuracy of the holographic acoustic field reconstruction. Therefore, this paper adopts a new parallel neural network structure incorporating inception and residual layers, which can obtain the sound pressure distribution information in the holographic acoustic field in multiple scales and prevent the problem of gradient disappearance or explosion. Considering the randomness of the focal point distribution and the uncertainty of the number of focal points in the holographic acoustic field, there will be locations in the holographic acoustic field where the acoustic pressure is 0, or the acoustic pressure is constant. The locations with constant sound pressure contain rich multi-focus information that needs extra attention, so the channel attention mechanism is introduced to focus on certain feature channels and enhance the network’s ability to extract multi-focus information.

We built a multiple regression network model (Res-Inception-ECA net, RIE-Net) incorporating an attention mechanism, and the overall framework of the model consists of three parts. The first part expands the original number of channels after four feature extraction operations and gradually compresses the original feature map width and height, which can provide deep abstract information through map features. Each time, the feature map passes through a convolutional layer followed by a batch normalization layer (BN) and a CeLU activation function. Considering the small scale of the focal information in the sound field, the convolutional layer kernel size is 1 × 1, which is used to balance the number of network parameters and the network feature extraction capability. After that, the feature maps are downsampled by a maximum pooling layer, which facilitates the reduction in feature dimensionality and increases feature invariance to input distortion.

The second part deepens the channels again after four feature fusion operations each time the feature map passes through an inverse residual layer [18] and inception layer [19], respectively. The first convolution of the inverse residual layer is a dimensionalization operation that expands the number of channels to extract more information on the high-dimensional space. The residual structure superimposes the target matrix directly onto the output, allowing the detailed information lost in the convolution process to be preserved. The Inception layer splices the target matrices processed by different convolution layers in terms of dimensionality, expanding the depth and width of the network and enhancing its adaptability to the input scale. The output feature map size and the number of channels of the inverse residual layer are kept the same as those of the inception layer. Considering the randomness and imbalance of the intensity information distribution in the sound field, the two similar feature maps generated are passed through an adaptive convolution kernel size channel attention mechanism layer [20] and a maximum pooling layer, respectively, and finally these two feature vectors are stitched together.

**Figure 4:**
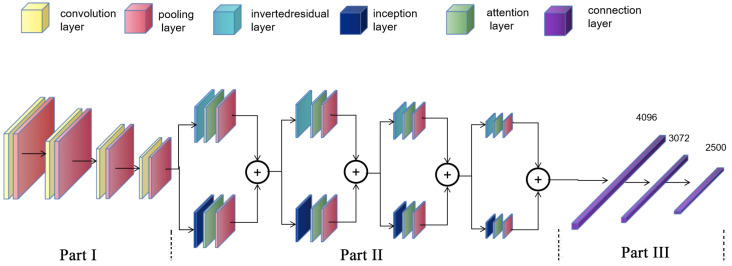
The framework of the proposed network model with an input dimension of 5 × 8000 × 1 and output dimension of 1 × 2500.

The first two parts of the model need to be properly structured and the hierarchy optimized to enhance performance. In the third part, the feature map is processed by the spreading layer and two fully connected layers with output dimensions of 3072 and 2500, which can generate a feature vector with an output dimension of 1 × 2500 to represent the phase of the transducer in the PTA. The overall structure of the model is shown in Figure 4.

The RIE-Net model uses the CELU activation function [21], as shown in Equation (4), where *x* is the input and *α* is the scale factor. It is continuously differentiable at all points, which not only does not encounter the problem of exploding or disappearing gradients, but also has higher accuracy, which makes the computational efficiency improved.

(4) $$\mathrm{CElu}\left(x\right)=\left\{\begin{array}{c} ɑ\left(e^{x/ɑ}-1\right)\;\;\;\;\;\;x<0\\ x\;\;\;\;\;\;\;\;\;\;\;\;\;\;\;\;\;\;\;\;\;\;\;\;\;x\geq 0 \end{array}\right.$$

### 3.2. Design of the Loss Function

Acoustic waves are periodic in nature, and the L1/L2 losses in traditional regression problems cannot be used directly in this physical context. In order to penalize the difference between the predicted phase value mean ($\varphi_{pred}$) and the true value mean ($\varphi_{truth}$), this paper designs the loss function by calculating the cosine of the difference between $\varphi_{pred}$ and $\varphi_{truth}$, as shown in Equation (5). The most important feature of this function is that the cosine operation can fully consider the periodicity of the acoustic wave.

(5) $$L=\frac{1}{N^{2}}\overset{\left(N,N\right)}{\underset{\left(u,v\right)=1,1}{\sum }}\left(1-\cos\left(2\pi \left(\left(\varphi_{u,v}\right){}_{pred}-\left(\varphi_{u,v}\right){}_{truth}\right)\right)\right)$$

where $\left(\varphi_{u,v}\right){}_{pred}$ is the predicted phase value of the transducer in the PTA, $\left(\varphi_{u,v}\right){}_{truth}$ is the true phase value of the transducer in the PTA, and $N^{2}$ is the number of transducers.

## 4. Physics Based Data Generation and Pre-Process

This section details the data set preparation and pre-processing methods in the deep learning scheme. Data acquisition is a necessary prerequisite for successful training of the network. The input to the neural network is composed of the location and sound pressure information of individual sampling points in the holographic acoustic field; the phase distribution of the transducer in the PTA is used as the truth value label for the network. The data pairs consisting of input data and true value labels are normalized to form a complete data set.

### 4.1. Generation of Data Sets

**Figure 5:**
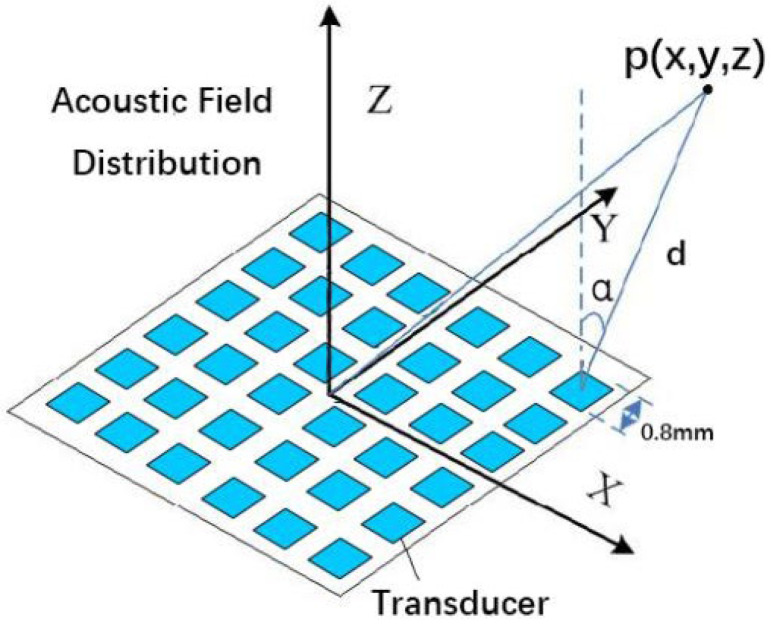
Model relationship diagram of the pressure generated by PTA at a sampling point in space.

There are two ways to obtain training datasets, namely physical measurements and simulated data. In this study, we aim to solve the problem of “backward propagation” of the holographic acoustic field. Because the simulated data have some advantages in solving the standardization problem, the forward propagation model is known and easy to compute, which is suitable for the rapid production of deep neural network datasets. Therefore, the simulation method was used to generate the dataset for the experiments. In the previous section, a geometric model relationship was established for the PTA-generated holographic acoustic field, and next, the numerical relationship between the parameters was described. Figure 5 shows a schematic diagram of a control point *p*(*x*, *y*, *z*) generated in space by the PTA.

Suppose an acoustic transducer *j* emits at a constant frequency and amplitude, $a^{j}$ is the amplitude of the transducer, $\varphi^{j}$ is the phase of the transducer, and $M^{j}$ is a complex number as a complex propagator from the position of transducer *j* to the point *r*. Then, the complex sound pressure $p^{jr}$ generated by the transducer at a point *r* can be modeled as:

(6) $$p^{jr}=a^{j}e^{i\varphi^{j}}M^{j}$$

For a known transducer and a point in space,$\;M^{j}$ is a constant. $M^{j}$ is usually calculated using several methods such as matrix method, finite difference method, or experimental measurements. In this paper we model the transducer emission as a rectangular single-frequency piston source [22] to calculate $M^{j}$. Thus, the transducer *j* generates a complex sound pressure $p^{jr}$ at point *r* which is in turn modeled as:

(7) $$p^{jr}=A\frac{D\left(\theta ,\beta \right)}{d}e^{j\left(\varphi_{m,n}+kd\right)}$$

The square piston source theoretical model [23] is used in this experiment. This theoretical approach directly solves forward propagation by treating each transducer as a point source or a square source using the cumulative method. For a PTA with multiple transducers, the total acoustic radiation pressure on the object is linearly superimposed by the acoustic radiation pressure from each transducer, so the total acoustic pressure field can be obtained by summing up the contributions of each source. The complex sound pressure *p*(*x*, *y*, *z*) generated by the PTA at a sampling point (*x*, *y*, *z*) is deduced as:

(8) $$p\left(x,y,z\right)=\overset{M,N}{\underset{m,n=0}{\sum }}A\frac{D\left(\theta ,\beta \right)}{d}e^{j\left(\varphi_{m,n}+kd\right)}$$

where *A* is a constant defined by the acoustic transducer power, which is kept consistent for all transducers. *M*×*N* is the number of transducers, and the coordinate positions are denoted by (*m*, *n*). *D*(*θ*, *β*) is the far-field directivity function based on the rectangular piston source model, which can be described as the product of the components of the wave vector on the two centerlines of the rectangle, and it depends on the polar *θ* and azimuthal angles *β* between the sampling point of the holographic acoustic field and the normal of the transducer. *d* is the Euclidean distance between the transducer and the sampling point. $\varphi_{m,n}$ is the initial emission phase of the transducer. *k =* 2*π*/*λ* is the wave number and λ is the wavelength of the acoustic wave (*λ = c*/*f*, wave velocity of *c =* 346 m/s in air at 25 °C and ultrasonic frequency *f =* 40 kHz).

The known information of each sample point in the holographic acoustic field includes coordinates ($x_{l}$, $y_{w}$, $z_{h}$) and sound pressure intensity $A_{x,y,z}$. The known information in the PTA includes transducer coordinates ($x_{m}$, $y_{n}$) and transducer emission acoustic wave amplitude $\left(A_{m,n}\right)$. However, the phase distribution ($\varphi_{m,n}$) of the transducer and the phase ($\varphi_{l,w,h}$) of the sampled points in the holographic acoustic field are unknown and unconstrained, and it is necessary to solve the unknown information to reconstruct the target acoustic field based on the known information. The PTA used in this experiment controls only the transducer phase (i.e., $A_{m,n}$ = 1, ∀(*m*, *n*) ∈ T). To generate meaningful samples of the dataset, the iterative backpropagation algorithm (IBP) is used to iteratively optimize the phase distribution of the PTA, and the computed results are used as the true value labels of the dataset. To clearly describe the computational process of IBP, let **S** denote the set of information about the sampling points (focal points or traps) in the holographic acoustic field, and **T** denote the set of phases of the transducers in the PTA arranged according to the above method.

The IBP algorithm treats the phase of the transducer in the PTA as the sum of the contributions from each sampling point in the holographic acoustic field. The calculation process is to first set the initial sound pressure phase of each sampling point in **S** to zero (i.e., $\varphi_{l,w,h}$ = 0) and then to back-propagate the solution to obtain the phase in **T**. After that, the phase information in **T** is brought into the forward propagation Equation (8) and solved to obtain the sound pressure phase of the sampling points in **S**. In this way, iterations are continuously cycled so that the inverse problem is solved to determine the phase of each point in the acoustic field. If the phase change in two consecutive iterations **T** is below a certain threshold, the algorithm stops, and the result is used as the transducer phase corresponding to the target holographic acoustic field is generated. The specific calculation process is shown in Algorithm 1.


**Algorithm 1:** Dataset Preparation**Data**: position, patterned phase, complex pressure, amplitude of transducer (*m, n*) is ($x_{m}$, $y_{n}$), $\varphi_{m,n}$, *p*($x_{m}$, $y_{n}$), and $A_{m,n}$; position, complex pressure, amplitude, phase of sampled physical point in acoustic field is ($x_{l}$*,*$y_{w}$*,*$z_{h}$), *p*($x_{l}$, $y_{w}$, $z_{h}$), *A_x,y,z_*, $\varphi_{l,w,h}$; an identity matrix *Il*,*w*,*h*, directivity function *D*(*θ*, *β*), wave number *k.* **Result**: transduer phases *φ*, complex acoustic pressure *p*(*xl*, *yw*, *zh*)1  *P*0←*Il*,*w*,*h*, *d*←[(*xl*−*x_m_*)2 + (*yw*− *y_n_*)2 +c*zh*2]0.5, *D*←*D*(*θ*, *β*), 0←$\varphi_{l,w,h}$, *H*←D×$e^{j\left(kd\right)}$/d2  iteration←0, ntrue←03  **while** iteration ≤ 200 and ntrue ≤ 2000 do4 *p*($x_{m}$, $y_{n}$)←∑*A_x_*_,*y*,*z*_*·ej*(*φ _l_*_,_*_w_*_,_*_h_*)*·*$H^{*}$;*#*$H^{*}$ *is the conjugate of* **H**.5 *p*($x_{m}$, $y_{n}$)←*p*($x_{m}$, $y_{n}$)/$\left|p\left(x_{m},\;y_{n}\right)\right|$∗*Il*,*w*,*h*;6 $A_{m,n}$←[[*p*$x_{m}$, $y_{n}$).real]2 + [ *p*($x_{m}$, $y_{n}$).img]2]^0.5^; 7 $\varphi_{m,n}$←(*p*($x_{m}$, $y_{n}$).img, *p*($x_{m}$, $y_{n}$).real);8 *p*(*xl*, *yw*, *zh*)←∑*P*0*ej*(*φm*,*n*)*·H*; 9 *p*(*xl*, *yw*, *zh*)←*p*(*xl*, *yw*, *zh*)/$\left|p\left(xl,\;yw,\;zh\right)\right|$*A_x,y,z_10 A_x,y,z_←[[*p*(*xl*, *yw*, *zh*).real]2 + [*p*(*xl*, *yw*, *zh*).img]2]^0.5^; 11 *φl*,*w*,*h*← (*p*(*xl*, *yw*, *zh*).img, *p*(*xl*, *yw*, *zh*).real);12**if** *φm*,*n* – *φ* ≤ π/100 **then** ntrue← nture + 1; **end**
13  iteration←iteration + 1; 14  **end**


To perform supervised learning, the dataset needs to be composed of the same data pairs as **(S, T)**. The sampled point information **S** in the holographic acoustic field is used as the network input, and the phase **T** of the transducer in the PTA is used as the network real value label. The input data structure is shown as **S** in Equation (9), which has an input dimension of 5 × 8000 × 1. The number of columns (*L* × *W* × *K*) of **S** represents the number of sampled points in the acoustic field, and each row in **S** is the polar coordinates (*ρ*, *θ*, *β*), sound pressure intensity (*A*), and sound pressure phase (*φ*) information of the sampled points in order from top to bottom. The size of the data set selected for this experiment is 20,000 groups, which are divided into test set, validation set, and test set in a completely random ratio of 17:2:1.

(9) $$S=\left[\begin{array}{c} \begin{array}{c} \rho_{1}\\ \theta_{1} \end{array}\\ \begin{array}{c} \beta_{1}\\ A_{1}\\ \varphi_{1} \end{array} \end{array}\;\;\begin{array}{c} \begin{array}{c} \rho_{2}\\ \theta_{2} \end{array}\\ \begin{array}{c} \beta_{2}\\ A_{2}\\ \varphi_{2} \end{array} \end{array}\ldots \ldots \ldots \begin{array}{c} \begin{array}{c} \rho_{L\times W\times H-1}\\ \theta_{L\times W\times H-1} \end{array}\\ \begin{array}{c} \beta_{L\times W\times H-1}\\ A_{L\times W\times H-1}\\ \varphi_{L\times W\times H-1} \end{array} \end{array}\;\begin{array}{c} \begin{array}{c} \rho_{L\times W\times H}\\ \theta_{L\times W\times H} \end{array}\\ \begin{array}{c} \beta_{L\times W\times H}\\ A_{L\times W\times H}\\ \varphi_{L\times W\times H} \end{array} \end{array}\right]$$

### 4.2. Data Pre-Processing

To improve the stability of the training process and enhance the model generalization, the input samples of the neural network are normalized to enhance the model generalization ability. In this experiment, a customized normalization method considering the physical background is applied to the data set. Each horizontal cross-section of the holographic acoustic field is treated separately so that the energy extremes of the sound pressure intensity are bounded in a reasonable interval. Values of sound pressure intensity greater than a certain threshold (*α*) and less than a certain threshold (*β*) will be reassigned as in Equations (10) and (11). Finally, all data sets are saved using the same format and size.

(10) $$\begin{array}{c} \alpha =2^{-0.25}max{\left(A_{x,y,z}{}_{\;}\right)}_{i}+\left(1-2^{-0.25}\right)min{\left(A_{x,y,z}{}_{\;}\right)}_{i}\\ \beta =2^{-0.25}min{\left(A_{x,y,z}{}_{\;}\right)}_{i}+\left(1-2^{-0.25}\right)max{\left(A_{x,y,z}{}_{\;}\right)}_{i} \end{array}$$

(11) $$A_{x,y,z}{}_{\;}=\left\{\begin{array}{c} \alpha ,\;A_{x,y,z}{}_{\;}>\alpha \\ \beta ,\;A_{x,y,z}{}_{\;}<\beta \\ A_{x,y,z},\;A_{x,y,z}{}_{\;} \end{array}\right.$$

where $A_{x,y,z}{}_{\;}$ is the sound pressure intensity, $max{\left(A_{x,y,z}{}_{\;}\right)}_{i}$ is the maximum value of sound pressure intensity in cross-section *i*, $min{\left(A_{x,y,z}{}_{\;}\right)}_{i}$ is the minimum value of sound pressure intensity in cross-section *i*.

## 5. Experiments

This section provides some experimental details and visualizes the experimental results of the proposed model in predicting the phase distribution of the PTA in a pictorial manner. We also discussed the results of the phase tests of the network model for individual and overall samples. Finally, the performance of the proposed model in solving the inverse mapping problem is evaluated based on the reconstruction of the holographic acoustic field.

### 5.1. Experiments Setup

**Figure 6:**
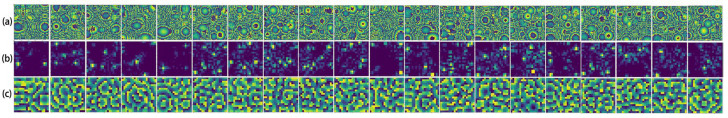
Partial training data pairs: (**a**) phase distribution of the transducer in the PTA; (**b**) a cross-sectional plot of the sound pressure intensity distribution in the holographic acoustic field; and (**c**) a cross-sectional plot of the sound pressure phase distribution in the holographic acoustic field.

The phase distribution of the transducer in the PTA and the sound pressure intensity and phase distribution of the sampled points in the holographic acoustic field can both be considered as grayscale images. Figure 6 shows an example plot of some of the data pairs, which are packed with COIs of different sizes and spatial locations for best generality.

The proposed model is trained on a RTX A5000 (24 GB) GPU server, a Window 10 operating system, and a Python 3.8 compiled environment, using PyTorch to build the deep learning framework. The initial learning rate of the proposed model is 0.002 for optimal parameter estimation, and the learning rate is automatically reduced by a factor of 0.98 for stagnation loss during training. The optimizer uses Radam [24], which has the advantages of both Adam and SGD, ensuring fast convergence and not falling into local optimum solutions easily. The model reaches full convergence after 120 rounds of training. To effectively quantify the metric of the difference between the predicted phase values and the true values, the loss function proposed in the previous section (i.e., Equation (5)) is used to measure the prediction accuracy of the model.

**Figure 7:**
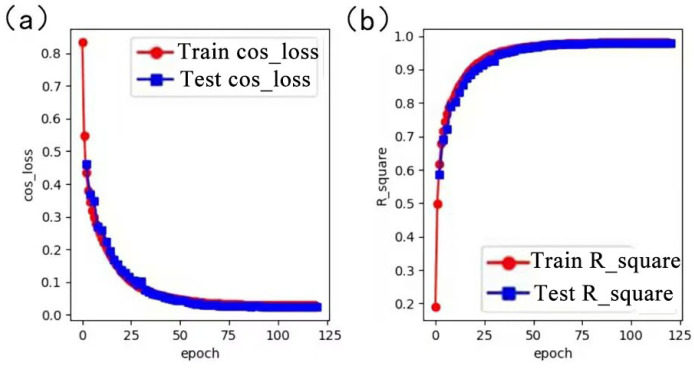
(**a**) Plot of loss function with the number of loop iterations during training and validation. (**b**) Plot of coefficient of determination with the number of loop iterations during training and validation.

Figure 7 shows the graphs of the training and validation process of the RIE-Net proposed model. The loss functions *train-COS_loss* and *val-COS_loss* are plotted with the number of loop iterations (*epoch*), and the coefficients of determination *train-*$R^{2}$ and *val-*$R^{2}$ are plotted with the number of loop iterations (*epoch*). The loss functions get smaller as the number of training iterations increases. The coefficients of determination become larger and larger as the number of training iterations increases. In each iteration, the *val-COS_loss* gradually approaches the *train-COS_loss*, and the proposed model is considered to be trained when the two values are approximately similar. The mean value of the error of the trained model is found to be stable at 0.025, and the $R^{2}$ of the fit is 0.98. The mean value of the error of the AcousNet method on the same test data set is about 0.05. Therefore, it shows that the RIE-Net method has higher prediction accuracy than the AcousNet method for the transducer in PTA.

### 5.2. Predictive Performance Analysis

**Figure 8:**
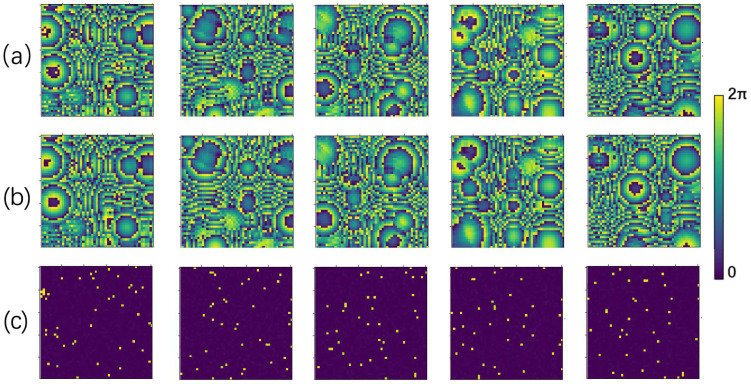
Partial prediction results (chromaticity bars at the right end of the figure are spectral bars and units (0, 2π) represent phases, where the hologram size is 5 × 5 ${\mathrm{cm}}^{2}$): (**a**) Ground truth values of the transducer phase distribution; (**b**) predicted values of the transducer phase distribution; and (**c**) plot of the difference between ground truth and predicted values of the transducer phase distribution.

Five samples were randomly selected from the test set to evaluate the learning performance of the RIE-Net neural network by comparing the difference between the predicted phase and the ground truth. Figure 8 shows the prediction performance of the neural network as an image (size 50 × 50). Figure 8a shows the ground truth phase of the transducer in the PTA, and Figure 8b shows the transducer phase obtained from the prediction of the neural network, whose high contrast illustrates the good performance of the RIE-Net neural network. Figure 8c shows the direct difference plot between the predicted phase and the ground truth, and the comparison shows that the difference is slight. The holographic acoustic field generated by the PTA is directly calculated by the forward propagation Equation (8), so it is completely feasible to achieve the reconstruction of the holographic acoustic field by using the neural network to predict the transducer phase in the PTA.

**Figure 9:**
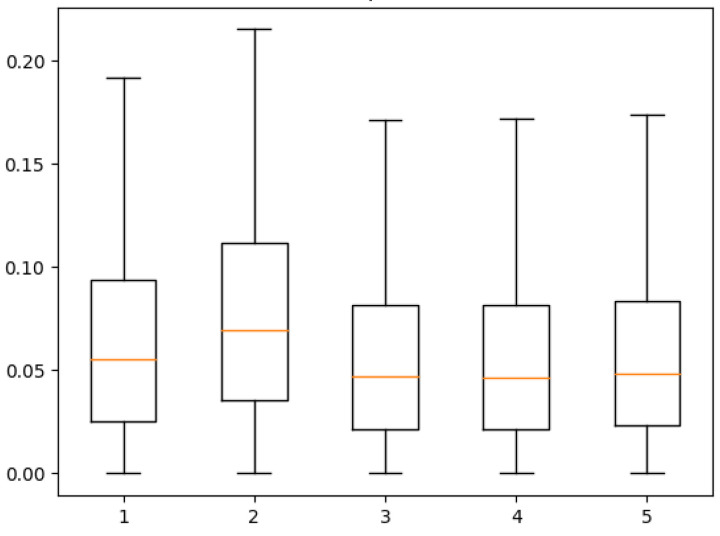
Prediction error quartile description of the RIE-Net neural network for five random samples (unit: Radian).

To further illustrate the accuracy of the RIE-Net neural network’s prediction results for a single sample, the prediction error ($2\pi (\left(\varphi_{u,v}{)}_{pred}-\left(\varphi_{u,v}\right){}_{truth}\right)$) of the neural network for the phases of the transducer (50 × 50) was experimentally tested. Figure 9 evaluates the box line plots for each of the five sample quartiles represented above. As seen from the data, the median prediction error of the RIE-Net method for all five samples is around 0.05 rad, while the median prediction error of the AcousNet method is around 0.1 rad, indicating that the RIE-Net method has a lower average error in the data prediction. Meanwhile, the RIE-Net method concentrates the data of each box with less error fluctuation, which indicates that the prediction stability is better than that of the AcousNet method. Considering the periodicity of acoustic waves, the phase differences of *θ* and 2*π-θ* are the same as each other, so the RIE-Net neural network not only achieves the phase prediction of the transducer in PTA but also maintains a high accuracy.

**Figure 10:**
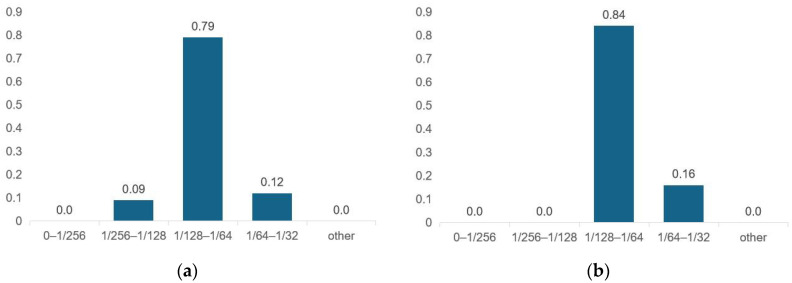
Statistical results of the overall prediction performance of the RIE-Net and AcousNet methods on the test dataset (**a**) RIE-Net; (**b**) AcousNet.

To measure the prediction performance of the RIE-Net neural network for the entire test dataset. The mean values of the phase errors ($\left(\varphi_{u,v}\right){}_{pred}-\left(\varphi_{u,v}\right){}_{truth}$) of the data set were statistically evaluated, and the results are shown in Figure 10. As seen in the figure, the average phase error of the RIE-Net method predicted data is no more than π/32, the prediction error accuracy of most of the data is between π/128 and π/64, and the highest error accuracy can reach between π/256 and π/128. Since the PTA device is driven by an FPGA [14], the phase interval is [0, 2π] discrete distribution, and the work of G. Memoli et al. illustrates that 4-bit phase coding is sufficient to produce a high-fidelity holographic acoustic field [25] with the corresponding phase coding resolution of π/8. Therefore, the performance of the proposed RIE-Net neural network is applicable in practical applications.

### 5.3. Real-Time Performance Analysis

The real-time performance of the holographic acoustic field reconstruction is crucial for the stability and controllability of the manipulation in practical applications, while the increase in control points in the acoustic field or the increase in the number of transducers may lead to an increase in its computation time. Therefore, the RIE-Net neural network is compared with the traditional iterative optimization algorithm-IB algorithm [14], and the results are shown in Table 1.

As can be seen from the table, to generate a simple multifocal acoustic field, the IB algorithm requires at least 15 min or more, while the RIE-Net network is computationally efficient and requires only 215 ms. Therefore, the RIE-Net neural network can quickly extract the phase information needed to reconstruct the target holographic acoustic field, eliminating the time-consuming mathematical iteration process, which is suitable for holographic acoustic fields with more control points or PTA devices with a large number of transducers.

### 5.4. Analysis of Holographic Acoustic Field Reconstruction Results

The predictive capability and real-time performance of the RIE-Net neural network were reasonably evaluated and discussed in the previous section, but the goal of this study is to generate a holographic acoustic field from PTA via a neural network. Therefore, in order to verify the accuracy of the acoustic field reconstruction results, this section uses the RIE-Net method to reconstruct the holographic acoustic field from the PTA and evaluates the prediction performance of the RIE-Net method by comparing the differences between the real and reconstructed holographic acoustic field.

**Figure 11:**
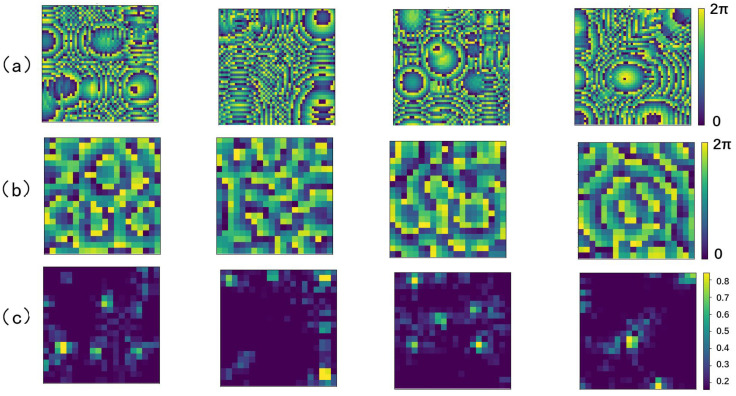
Cross-sectional view of the holographic acoustic field generated by PTA in a plane, where the hologram size is 5 × 5 ${\mathrm{cm}}^{2}$ the chromaticity bar at the right end of the figure is the spectral bar, and the unit (0, 2π) represents the phase. (**a**) Phase distribution of the PTA; (**b**) acoustic pressure phase distribution of the holographic acoustic field in the plane z = 1.25 mm; (**c**) acoustic pressure intensity distribution of the holographic acoustic field in the plane z = 1.25 mm.

Four randomly selected sample data pairs from the test set are presented in image form, as shown in Figure 11. The data pairs specifically include the phase distribution of the PTA, as well as the sound pressure intensity and phase of its corresponding holographic acoustic field at a certain horizontal cross-section, where the sound pressure intensity is normalized for better comparison.

**Figure 12:**
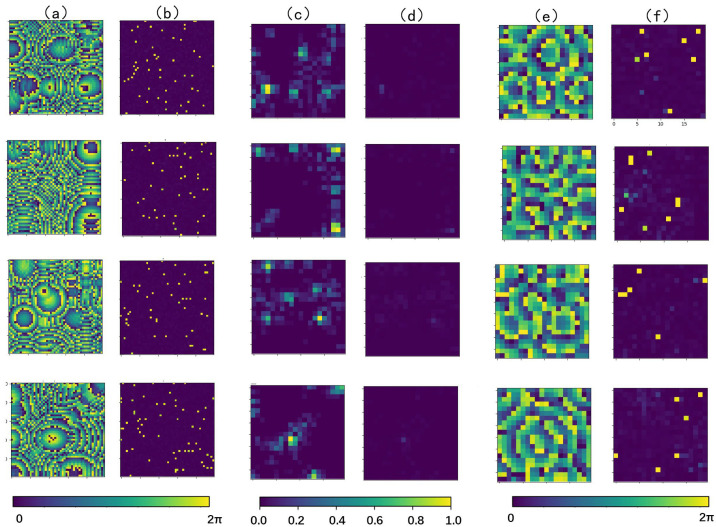
A cross-sectional screenshot of the holographic acoustic field generated by the RIE-Net method in a plane (*z =* 1.25 mm) with a hologram size of 5 × 5 ${\mathrm{cm}}^{2}$. The lower chromaticity bar in the figure is the spectral bar, and the units (0, 2π) represent the phases. (**a**) PTA phase distribution generated by the RIE-Net method; (**b**) predicted phase error of the RIE-Net method; (**c**) acoustic pressure intensity distribution of the simulated holographic acoustic field; (**d**) difference in acoustic pressure intensity distribution between the simulated and real holographic acoustic field; (**e**) acoustic pressure phase distribution of the simulated holographic acoustic field; (**f**) difference in acoustic pressure phase distribution between the simulated and real holographic acoustic field.

In order to evaluate the quality of the holographic acoustic field generated from the PTA based on the RIE-Net method, it is necessary to reconstruct the holographic acoustic field. The reconstruction process is as follows: first, four sets of multi-focus holographic acoustic field information are randomly selected from the test set as input samples, then they are put into the RIE-Net neural network for prediction, and the predicted phase values of the four PTAs are obtained, and finally the predicted results are used to generate a simulated holographic acoustic field from the PTA using the forward propagation Equation (8). The experiments are shown graphically to obtain the holographic acoustic field using the RIE-Net method. Figure 12 shows the sound pressure intensity and phase distribution of the simulated holographic acoustic field in a certain plane, and the difference between the simulated field and the real holographic acoustic field, where the sound pressure intensity is normalized for better comparison.

Next, we evaluate the accuracy of the reconstruction results. The above results show that the transducer phase (Figure 12a) obtained by the RIE-Net method fully supports the PTA generation of the corresponding holographic acoustic field (Figure 12c,e). The difference plots of sound pressure intensity and phase between the simulated and real holographic acoustic field were compared (Figure 12d,f), from which it can be seen that the error between the two is small, and the similarity is high. Meanwhile, the mean value of the structural similarity index (SSIM) between the simulated and real sound intensity distribution maps is 0.92, and the mean value of the peak signal-to-noise ratio (PSNR) is 29.76 in all test sets [26]. Therefore, the RIE-Net method proposed in this paper accurately predicts the phase distribution of the transducer in the PTA, which can not only reconstruct the contour and detail information of the original sound field quickly and efficiently but also maintain a high accuracy to the target.

## 6. Conclusions

The RIE-Net neural network proposed in this paper is a CNN-based regression network that can be used to compute the phase of the transducer in the PTA corresponding to the reconstructed holographic acoustic field. We train the network on the dataset generated by the simulation method and test the samples to demonstrate the reconstruction capability of the method. The simulation results show that the proposed method achieves higher real-time performance compared to conventional holographic acoustic field reconstruction methods. Additionally, the network has a higher accuracy when generating a holographic acoustic field based on the PTA compared to the latest AcousNet method. These promising results demonstrate the potential of deep learning methods in improving the accuracy and real-time performance of holographic acoustic field reconstruction. In future work, new methods based on deep learning will be further explored to enhance the performance of the proposed method.

## Figures and Tables

**Table 1:** Real-time performance comparison of RIE-Net method and IB algorithm.

FIELD TYPE\METHOD	RIE-NET	ACOUSNET	IB METHODS
**SINGLE-FOCUS**	215 ms	218 ms	15.4 min
**DUAL FOCUS**	215 ms	218 ms	16.6 min
**THREE FOCUS**	215 ms	218 ms	17.2 min
***COSINE LOSS ERROR***	0.025	0.05	/

## Data Availability

Data sets cannot be made public for copyright reasons.
